# Farm-Specific Effects in Predicting Mastitis by Applying Machine Learning Models to Automated Milking System and Other Farm Management Data

**DOI:** 10.3390/ani15192825

**Published:** 2025-09-28

**Authors:** Muhammad N. Dharejo, Olivier Kashongwe, Thomas Amon, Tina Kabelitz, Marcus G. Doherr

**Affiliations:** 1 Institute for Veterinary Epidemiology & Biostatistics, School of Veterinary Medicine, Free University of Berlin, House 21, Königsweg 57, 14163 Berlin, Germany; 2 Department of Sensors & Modelling, Leibniz Institute for Agricultural Engineering and Bioeconomy, Max-Eyth-Allee 100, 14469 Potsdam, Germany; 3 Institute for Animal Hygiene and Environmental Health, School of Veterinary Medicine, Free University of Berlin, Robert-von-Ostertag-Straße 7-13, 14163 Berlin, Germany

**Keywords:** mastitis prediction, machine learning models, automatic milking system, time series data, farm-specific effects

## Abstract

Early and accurate prediction of mastitis is crucial in effective herd management and minimizing economic losses. This study investigated the effects of farm-specific factors on the accuracy of mastitis predictions by applying machine learning (ML) models to an automated milking system (AMS) and farm management data. We analyzed a large dataset consisting of 5.88 million observations over the period of 2019–2024 from four dairy farms in Germany. Six ML algorithms were applied to predict mastitis occurrence, with a focus on understanding how farm-specific factors like herd size, management practices, and farm environment may influence prediction accuracy. For training and testing on combined data, the accuracy, sensitivity and specificity ranged between 83 and 92%, 78 and 93% and 83 and 92%, respectively, with an area under curve (AUC) between 91 and 96%. However, under mixed-to-individual farm effects analysis, results exposed weaknesses in the generalization. Models adapted well to internal patterns when analyzing each individual farm separately, reaching very high AUCs of up to 98%, but the results were significantly different again when analyzed with a leave-one-out approach. The analysis determined that data from each farm carries variable underlying patterns, suggesting that a tailored approach to each farm’s unique characteristics might improve mastitis prediction through ML.

## Simple Summary

Mastitis is a common disease in dairy cows that can cause major losses for farmers. Predicting it early can help prevent problems. This study used computer programs called ‘machine learning models’ to see how well they could predict mastitis in cows using data from automatic milking robots and farm records, with a focus on differences between farms. We looked at information from four farms in Germany, covering nearly 6 million records from 2019 to 2024. The machine learning models were pretty accurate overall—in many cases, they predicted mastitis correctly around 83–92% of the time. But the accuracy changed depending on which farm the data came from. Each farm had its own unique setup, like different herd sizes and management styles. When the models were tested on combined data from all farms and on the data of each individual farm, they worked well—but when trying to predict mastitis on a farm not included in the dataset for training the models, the results were not as good. This shows that each farm is different, and using a one-size-fits-all model might not work. To achieve the best results, it is better to customize the prediction model to each farm.

## 1. Introduction

Mastitis, an intra-mammary infection (IMI), is the most common and economically important disease in dairy cattle, typically resulting in decreased milk production and quality. Furthermore, mastitis is often associated with pain and discomfort that can negatively impact animal welfare [1,2]. Mastitis is the most frequently occurring disease, ranging from 14.2 to 16.3 percent of herds, depending on the region, in Germany [3]. Traditional mastitis detection methods, i.e., the regular physical examination of cow teats and milk and laboratory testing, as well as being time-consuming and labor-intensive, are becoming less practical due to the rise of fast-paced milking operations with automatic milking systems (AMSs). Therefore, digitized early detection and management of mastitis are crucial in maintaining animal health, optimizing milk yield, and minimizing financial losses for dairy farmers [4].

The entire concept of dairy farming has changed since the adaptation of AMSs because of its ability to continuously monitor individual cows’ performance by collecting a vast array of data on milk production and quality. These systems offer a unique opportunity for high-resolution data aggregation, which, despite its complexity, may enhance mastitis detection through the integration of advanced data analytics [5,6]. Machine learning (ML) models, with their capacity to handle large and complex datasets, have demonstrated considerable promise in predicting health conditions in dairy cows [7]. Taking advantage of the real-time data generated by AMSs, it is possible to develop ML models that can correctly predict mastitis events by identifying early signs through changes in data patterns. The development of such predictive models is likely to enable timely interventions [8]. Researchers have been searching for modeling strategies that show greater performance in predicting mastitis, but there is a need to achieve further progress by creating models that have higher prediction rates [9].

A number of studies have determined the risk of mastitis associated with farm management practices that can partly explain the variance in mastitis cases on farms [10]. Factors including increased exposure to environmental pathogens, dietary components, genetic traits, and the introduction of new stock into an established herd have been reported to be correlated with mastitis occurrence [11,12,13]. Farmers’ attitudes and the type of mastitis-causing pathogens have an impact on the rate of mastitis incidence and somatic cell counts on respective farms [14]. Most ML models developed for mastitis prediction so far do not sufficiently consider variability between farms, which can impact model accuracy and generalizability. Farm-specific factors such as herd size, management practices, environmental conditions, and milking equipment calibration could influence the incidence and characterization of mastitis. As such, there is growing recognition that ML models must be tailored or adjusted to specific farm conditions to enhance their predictive performance [15].

The aim of this study was to examine how farm-specific characteristics, as captured through AMS data, together with other farm-related variables, affect the ability of ML models to predict mastitis, with particular attention given to the challenges posed by variability and heterogeneity in data patterns observed across different farms. The specific objectives were to identify the differences in mastitis prediction by ML models by exploring the following scenarios:

(1) Combined-farm effects (evaluating ML models on pooled data from all farms);
(2) Combined-to-individual farm effects (evaluating generalization ability of models for individual farms);
(3) Individual-farm effects (understanding model performance within a farm and comparing it between farms);
(4) Farm-to-farm effects (evaluating model generalization to yet unobserved farms).

The conclusions of this study will be used to propose approaches to implementing ML-based mastitis prediction algorithms in dairy herd-health management systems.

## 2. Materials and Methods

Cow-level sensorial data generated by an AMS, as well as animal health and other cow-related farm-specific data extracted from farm management records, along with climate data, were processed and included in machine learning applications. Ethics approval was not required for this study because data recording had already taken place as part of routine farm operations, and these data were extracted from the stored electronic farm records with the consent of the respective farmers.

### 2.1. Data Collection and Preprocessing

Time series data for individual cow milking recorded by an AMS, as well as animal health and other cow-related farm-specific data, were extracted from farm management records, and climate data from sensors on farm premises were used in this study to train and test the ML models for the prediction of mastitis events. The data were collected from four dairy farms located in two different federal states of Germany. The basic details of the participating dairy farms can be seen in Table 1.

The dataset from AMS records covered a period of five years and five months from 1 January 2019 to 31 May 2024 and contained a total of 5.88 million observations on daily milking of 4139 individual dairy cows. Seven predictor (x) variables were selected from the AMS data: electrical conductivity (EC), somatic cell count (SCC), milk yield (MY), milking flow (MF), milk temperature (MT), fat content in milk (FM), and protein content in milk (PM). All variables were recorded every time a cow was milked, ranging from two to four times a day. The date and time format of AMS data were YYYY/MM/DD and hh/mm/ss. The other cow-related input variables included were the number of lactations (NL) and days in milk (DIM). The climate data contained information on daily environmental temperatures (ET) and humidity (EH) with a time resolution of every ten minutes.

The mastitis event information was obtained from the animal health records of the farm management program. The outcome (y) variable was the status of a cow on the date of each observation. A cow was considered negative unless it received a treatment against mastitis prescribed by a veterinarian, in which case, the status became positive for the observations on that particular date of treatment. The treatment criteria against mastitis were based either on visible clinical signs or milk tests during routine checks. The date format of health record was YYYY/MM/DD, but the time of the treatment was not registered. All farm-specific data sources provided a specific identification number for each individual cow that allowed us to link records date-wise from different sources on the same individual cow. All data processing, modeling, and visualization were conducted by using 3.6.0. version of Python [16] using the libraries NumPy, pandas, seaborn, matplotlib, and scikit learn.

### 2.2. Data Processing

To merge the data from all sources, the time resolution of AMS data was changed to one time per day by taking average daily values of all predictors. The health and other cow-related information was merged with AMS data on the date of observation and the individual cow identification number by applying the inner merge option, i.e., merging only those data points that matched the exact date and identification number from all data sources. However, after taking daily mean values, the climate data were merged with the AMS data by the date of observation in both datasets. Missing values in AMS and climate data were marked as NaN (not a number) values during the process of taking daily means. The duplicate data points resulting from the merging process were removed based on individual cow identification and the date of each observation. The treatment events consecutive to the first record were considered duplicate positive cases and removed along with the corresponding AMS observation for up to two weeks after the date of initial treatment of each mastitis event. Therefore, all remaining positive observations were meant as new occurrences [17,18]. Further data processing approaches carried out in this study are described stepwise below.

### 2.3. Data Transformation

The dataset was transformed to include lagged information of all predictor variables from AMS data for up to four previous days by applying the autoregressive form of order p, AR(p) [19]. ML models showed the highest mastitis prediction rates when applied on transformed datasets containing three to four previous days of milking records [15,20,21]. After transformation, each row of the dataset contained (t + p) observations for all predictor variables from AMS data, as denoted in equation [1].

Xn(t) + Xn(t − 1) + Xn(t − 2), … + Xn(t − p),(1)

Here (X) represents the numerical input of predictor variables, (n) represents the total number of predictor variables, (t) represents the time series observation of the current day, and (p) represents the total number of time series observations from previous days before the current (t) observations (here, p = 1, 2, 3, and 4).

### 2.4. Procedure of ML Application

#### 2.4.1. Data Splitting

There are multiple procedures in practice to split a dataset, such as hold-out, K-folds, and stratified K-folds. It is important to prevent information leakage from the future to the past in time series data [22]. Keeping in mind our hypothesis and need to oversample the minority class, this study applied the hold-out method for training and testing the models. However, to correspond with each scenario in the study aims, the dataset was manually split into training and testing subsets based on time periods, as described below.

(1) Combined training and testing (mixed-farm effects): ML models were trained on combined data of all four farms for the period of 2019–2022 and tested on combined data of all farms for the year 2023 and up to May 2024.
(2) Combined training and individual testing (mixed-to-individual farm effects): ML models were trained on combined data from all farms for the period 2019–2022 but tested on individual data of each farm for the year 2023 and up to May 2024.
(3) Individual training and testing (individual-farm effects): ML models were trained on the data of each individual farm separately for the period from 2019 to 2022 and tested separately on each farm’s own data for the year 2023 and up to May 2024.
(4) Farm-to-farm training and testing (farm-to-farm effects): ML models were trained on the complete data of three farms but tested on the complete data of the fourth farm that was not included in the training of models (leave-one-out cross validation). The same procedure was applied for all four farms.

#### 2.4.2. Cross Validation and Hyperparameter Tuning

We chose the fine-tuned hyperparameters from a companion study that applied stratified 5-fold cross validation to a dataset of a similar nature by applying GridSearchCV [20]. However, to adapt it for the current data, we further fine-tuned the hyperparameters by applying RandomizedSearchCV. GridSearchCV is mostly used because of its simplicity and ease of application for large datasets [23]. In contrast to GridSearchCV, which is more exhaustive and requires a lot of time and computing resources, RandomizedSearchCV tries only a small subset of parameters from chosen distributions, and the model parameters are optimized by a cross-validated search across a range of choices [24]. Further details of the model parameters are given in Table 2.

#### 2.4.3. Resampling Technique

The original dataset was by nature highly dominated by the negative class in comparison to the positive one. Details of overall and farm-wise observations are presented in Table 3.

Classification models are more likely to adapt the algorithms of the dominant class and can be inclined towards the majority class while predicting. The ML classifiers may not rightly identify the rare events but could wrongly classify them as negative and still maximize the overall accuracy [25]. Therefore, after splitting the dataset into training and testing portions, the synthetic minority oversampling technique (SMOTE) was applied to the training data. The decision to apply SMOTE was based on the fact that it does not reduce the sample size of the majority class; hence, there is no risk of losing information. This technique creates synthetic examples by taking the line segments of k-nearest neighbors of the minority-class samples. The number of k-nearest neighbors is randomly chosen depending on the amount of oversampling required [26]. After oversampling, the number of positive events became exactly equal to the negative events in the training subset of this study. However, the test subset was not oversampled and represented the original proportion of negative and positive observations.

### 2.5. ML Models Evaluated

#### 2.5.1. Logistic Regression (LR)

The LR method estimates the parameters using the maximum likelihood estimation technique. It uses a set of input variables to calculate the probability of a discrete result, assuming that all of the input features are independent. In the health sciences, LR is frequently employed to ascertain whether an event occurred or not [27].

#### 2.5.2. Support Vector Machine (SVM)

SVM is a type of supervised machine learning. Its decision function defines an optimal hyperplane that leverages feature input information to distinguish one class from another. This hyperplane is then used to accurately classify the labels of new data. Linear SVM adheres to two types of margin guidelines: hard-margin and soft-margin. The hard-margin approach prohibits any incorrect classifications during training and is susceptible to overfitting. In contrast, the soft-margin approach is more forgiving of errors and permits misclassifications during training [28].

#### 2.5.3. Decision Tree (DT)

The DT classifier operates as a sequential flow using a structure that resembles a tree. It consists of three fundamental components: decision nodes, branches or edges, and leaves. Among various algorithms, ID3 and its successor C4.5 are the most widely used in decision-making. The operation of a decision tree includes three stages: selecting features, determining a partitioning strategy, and deciding when to stop partitioning [29].

#### 2.5.4. Random Forest (RF)

The RF classifier combines tens to thousands of individual decision tree classifiers. To ensure the necessary structural diversity among the decision tree bases, stochastic methods are utilized at two levels. Initially, each decision tree is generated on a uniformly sampled subset of the training dataset using a bootstrap method. Next, the potential splits are restricted to a randomly chosen subset of available candidates [30].

#### 2.5.5. Gradient-Boosting Decision Tree (GBDT)

GBDT is another ensemble that consists of base decision trees. The GBDT approach creates multiple decision tree learners by fitting the gradients of the residuals from the previously built tree learners. It reduces the chosen loss function of the new weak learner by carrying out regression on a function derived from the gradient vector of the loss function assessed in the prior iteration [31].

#### 2.5.6. Multi-Layer Perceptron Neural Network (MLP-NN)

This comprises three layers: an input layer, a hidden layer, and an output layer. The operation of MLP-NN relies on a supervised machine learning method known as back-propagation to train the network [32]. Due to non-linear boundaries, MLP-NN has an edge over traditional approaches. The number of hidden layers and the nodes per layer can be adjusted according to the modeling requirements. Generally, the higher the number of nodes, the greater the sensitivity of the model being applied, but the possibility of overfitting is also greater [33].

## 3. Results

### 3.1. Mixed-Farm Effects (Training Combined and Testing Combined)

The mastitis prediction results under mixed-farm effects (Table 4) indicated an overall accuracy between 83% and 92%. The sensitivity ranged between 80% and 93%, and the specificity ranged between 83% and 92%. The SVM classifier obtained the highest accuracy and specificity, followed by GBDT. The best sensitivity was shown by MLP-NN; however, its accuracy and specificity remained the lowest of all six ML models. The AUC of all models remained above 90%, with DT performing the lowest and RF, MLP-NN, and GBDT showing the highest AUC.

**Figure 1:**
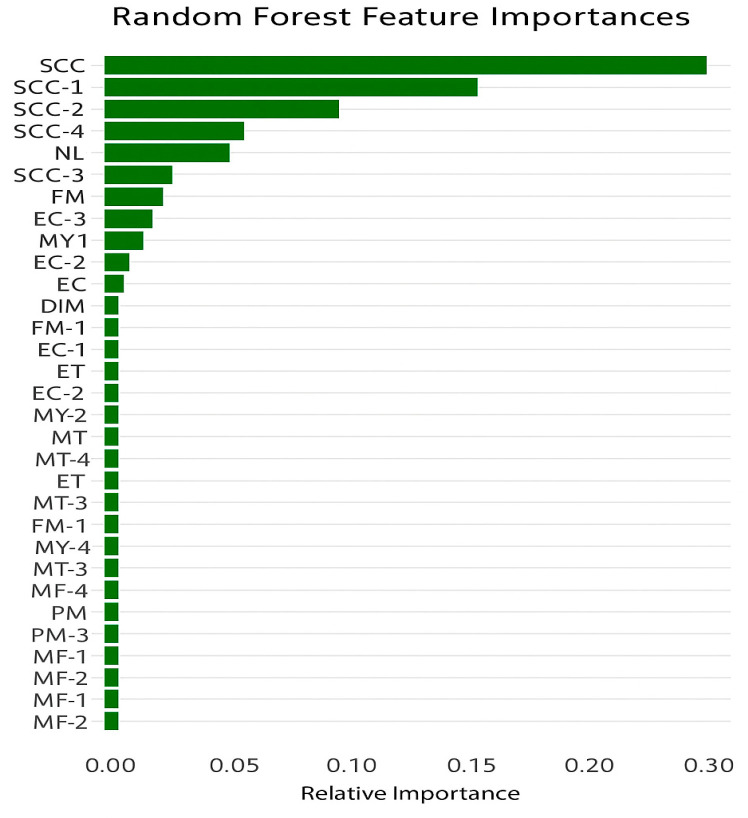
Relative feature importance of predictor variables (SCC, somatic cell count; NL, number of lactations; MY, milk yield; FM, fat content in milk; EC, electrical conductivity; DIM, days in milk; ET, environmental temperature; MT, milk temperature; MF, milk flow; PM, protein content in milk).

Relative feature importance calculated with the RF model indicated that all predictor variables had higher-than-zero relative importance (Figure 1). SCC values of the current day and previous days showed the strongest relativeness, followed by NL and MY. FM, EC, DIM, and ET showed comparatively moderate importance. PM and MF were relatively at the least level of importance.

**Figure 2:**
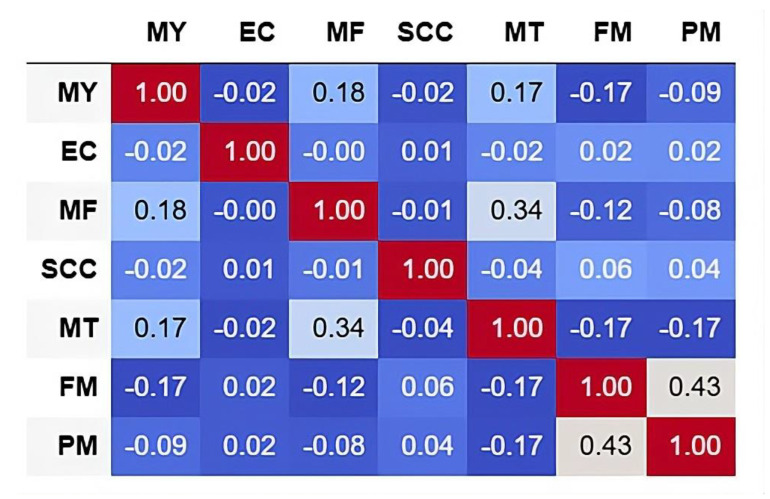
Feature correlation matrix of milking parameters (MY, milk yield; EC, electrical conductivity; MF, milk flow; SCC, somatic cell count; MT, milk temperature; FM, fat content in milk; PM, protein content in milk).

Furthermore, the feature correlation matrix indicated that the dataset’s features were fairly independent, with only a couple of moderately related pairs (Figure 2). The strongest meaningful relationship between FM and PM was moderately positive. MF and MT also showed slightly positive correlation. EC seems independent of other variables, whereas negative correlations between a few variables were not strong enough to imply any inverse relationship.

### 3.2. Mixed-to-Individual Farm Effects (Training Combined, Testing Separate)

The mastitis prediction results under mixed-to-individual farm effects (Table 5) indicated significant variations between the farms. Overall, the SVM classifier obtained the highest accuracy, specificity, and AUC. The MLP-NN model achieved the highest sensitivity for all farms. For farm B, all models have high AUCs ranging between 94 and 96%, and LR achieved the best accuracy. The results for farm G indicated the best accuracy, specificity, and AUC using the SVM model, and MLP-NN scored highest for sensitivity. Farm H indicated lower performance across all models, with a significant drop in accuracy and AUC. The LR and SVM models showed the highest accuracy, specificity, and AUCs for Farm M.

### 3.3. Individual-Farm Effects (Training and Testing Separately)

The results of individual-farm effects (Table 6) indicated different performances across the models and farms. For Farm B, RF and GBDT scored highest in accuracy and specificity with excellent AUC values. All models performed well for Farm G, with AUC ranging between 94 and 98%, and the MLP-NN had exceptionally well-balanced metrics. Farm H overall showed the weakest mastitis prediction results across the models, with the highest accuracy, specificity, and AUC achieved by RF and the highest sensitivity achieved by the MLP-NN model. All models were consistent with high prediction scores in the analysis of Farm M.

### 3.4. Farm-to-Farm Effects (Training on Data of Three Farms and Testing on Data of Fourth Farm)

Table 7 shows the accuracy, sensitivity, specificity, and AUC estimates of farm-to-farm effects for all four farms. SVM and LR models achieved the highest accuracy and specificity, but MLP-NN had the highest sensitivity for Farm B. Farm G showed excellent generalizability for all models with high prediction metrics. Farm H indicated the weakest prediction performance across all models with the highest AUC, up to 85%. Farm M indicated strong generalizability across the models with an AUC ranging between 90 and 96%.

## 4. Discussion

This study analyzed farm-specific effects in predicting mastitis by applying six ML models to data from AMSs and other farm management data collected from four dairy farms in Germany. To handle the issue of class imbalance, the positive class, being in the minority, was oversampled in the training portion of the dataset by using the SMOTE method. We applied four different training and testing approaches to observe the impact of farm variability in the patterns of each individual farm’s data on the mastitis prediction scores of the ML models. The criteria for the inclusion of farms were based on the availability of digital records of mastitis treatment, AMSs, and other farm-related data, as well as the willingness of farmers to participate in this study. The participating farms included one research farm and three commercial dairy farms of varying sizes. A population-representative sample size study in Germany found that a little less than 20% of dairy farms were equipped with an AMS, whereas around 80% were still using classic milking systems or conventional methods [34]. Despite the small number of farms in our study, we feel comfortable that these represent at least those farms in Germany (typically with more than 50 milking cows) that use an AMS.

The results of this study indicated that models performed well regarding combined training and testing scores due to the pooled data, but training in combination and testing separately exposed the weakness in generalization of the models. Similarly, the models adapted well when trained and tested on each individual farm but again showed limitations when trained on three farms and tested on unseen data from the fourth farm. Overall Farm H underperformed and Farm G performed the best. All ML models performed well in general, but each model showed its own strengths and weaknesses.

For mixed-farm effects, we applied ML models to the combined data of all farms. The best accuracy, sensitivity, and specificity scores reached 92%, 93%, and 92%, respectively, and the AUC across models ranged from 91 to 96%. At this stage, models generally performed well because the hyperparameters were fine-tuned on pooled data. However, under mixed-to-individual farm effects analysis, the results exposed weaknesses in generalization. For the analysis of individual-farm effects, to some extent, the models adapted well to internal patterns, reaching very high AUCs of up to 98%. Furthermore, in analyzing the farm-to-farm effects, we found that once again, the model prediction performance varied significantly among all farms.

Farm-wise results indicated weakness in the performance of Farm H, with the lowest sensitivity and an AUC as low as 74%, which could be due to a different AMS, as the other three farms used AMSs from the same manufacturer. Despite having the lowest percentage of positive class, Farm B adapted well to some models, reaching AUCs between 93 and 96%. Farms G and M showed excellent adaption to generalization, as well as in individual testing, reaching an AUC of up to 98% and 96%.

Regarding model performances, LR was consistent with a high AUC of up to 98% and performed well with generalization but with lower sensitivity rates. SVM achieved top AUCs with a balanced overall performance, but it also struggled with sensitivity for Farm H. DT was simple and fast but produced lower AUCs and sensitivity levels. The RF model was good with generalization, with strong AUCs and specificity rates, but its sensitivity levels were lower than those for MLP-NN, which showed the highest sensitivity scores, up to 97%, and it excelled in finding true positives. However, MLP-NN had lower specificity rates in comparison to other models. GBDT was competitive in some tests, with high AUCs, but at the same time, it showed moderate sensitivity metrics.

Among peers, the majority of studies have either relied on single-farm data or have rarely considered farm-to-farm differences in their analysis. Even though some researchers have reported farm-level differences in their mastitis prediction studies, the main focus has been on the overall predictive performance of ML models. Researchers in Canada applied a recurrent neural network (RNN) model on AMS data from a large number of herds and reported considerable decline in scores when the model was tested on data from new herds that were not included in the training set and attributed this difference to herd-specific variability [15]. Similarly, Australian scholars applied generalized logistic linear models (GLLMs) to AMS data collected from two pasture-based dairy farms in Australia and reported slight differences in sensitivity and specificity between the farms [35]. A study analyzed automated mastitis detection on two dairy farms located in Duiven and Lelystad, Netherlands. They found that the model had a significantly higher specificity level for the farm in Duiven compared to that in Lelystad [36].

The findings of this study have confirmed the impact of farm-level heterogeneity in AMSs and other farm-specific data in predicting mastitis through the application of ML models. It can be inferred that a successful mastitis prediction model at one farm may not be equally efficient in predicting mastitis on other farms. Hence, the generalizability of such models will face challenges during practical implementation when exposed to unseen data from new herds. Therefore, linking these models with a larger database and including data from each new farm in the training set may likely represent an advantage.

Although this study used a large dataset developed from AMS milking records and other farm variables, we acknowledge that more variables with further farm-specific information could be included if the data were available. Hence, this study recommends the inclusion of data from cow genetics, feed intake, health and hygiene interventions on the farm, and seasonal trends to fully explore the subject under investigation. The limitation in including more variables into such models, however, is that most variables typically need to undergo time-consuming preprocessing steps before they can be offered to the ML algorithm. This reduces the possibility of developing a practical “real-time” prediction tool to be implemented into herd-health management systems.

## 5. Conclusions

We analyzed farm-specific effects on mastitis prediction by applying six ML models to AMSs and other farm management data from four dairy farms in Germany. Our findings suggested that data from each farm had its own specific effects in mastitis prediction, and each ML model exhibited its own strengths and weaknesses depending on the scenario explored. It further implied that training ML models on combined data from all available farms might not allow them to adapt well to the farm-specific patterns in the data from each individual farm, especially when farms have AMSs from different manufacturers. We recommend fine-tuning models for each individual farm separately in addition to larger database of farms with similar AMS suppliers for comparative analysis and further cross validation.

## Figures and Tables

**Table 1:** Overview of basic details about four dairy farms participating in this study.

Farm Name	Farm B	Farm G	Farm H	Farm M
German Federal State	Brandenburg	Brandenburg	Brandenburg	Saxony
Farm Size (ha)	1200	940	3590	1370
Herd Size	220	230	560	820
AMS Type	Lely Astronaut *	Lely Astronaut	GEA Mione **	Lely Astronaut
Average Daily Milk Yield per Cow (L)	31	30	29	31

* Lely, Maassluis, The Netherlands; ** GEA, s-Hertogenbosch, The Netherlands.

**Table 2:** ML models and hyperparameters evaluated with RandomizedSearchCV.

ML Model	Method	Hyperparameters	
		Tested	Selected
Logistic Regression	Binary	C = range (1 to 30)	C = 10
Support Vector Machine	Linear	C = range (1 to 30)	C = 10
Decision Tree	Gini	Max depth: range (1 to 25)	Max depth = 12
Random Forest	Gini	Number of estimators: 5 to 50 Max depth: 5 to 20	Number of estimators = 25 Max depth = 12
Gradient-Boosting Decision Tree	Log loss	Number of estimators: 5 to 50 Max depth: 5 to 20	Number of estimators = 25 Max depth = 12
Multi-Layer Perceptron Neural Network	Input: Relu Output: Sigmoid	Hidden layer sizes: 10 to 50	Hidden layer sizes = 20

**Table 3:** Details of overall and farm-wise numbers of total, negative, and positive observations.

	Total Observations	Negative	Positive	Positive %
Overall	1,886,947	1,875,568	11,379	0.60
Farm B	282,291	281,921	370	0.13
Farm G	297,073	295,964	1109	0.37
Farm H	395,622	394,428	1194	0.30
Farm M	911,961	903,255	8706	0.95

**Table 4:** Mastitis prediction estimates of six ML models after being trained and tested on the combined data of four dairy farms.

Farm	ML Models	Accuracy (%)	Sensitivity (%)	Specificity (%)	Area Under Curve (%)
All	LR	91	83	91	95
	SVM	92	81	92	95
	DT	89	80	89	91
	RF	89	87	89	96
	MLP-NN	83	93	83	96
	GBDT	92	78	92	96

**Table 5:** Mastitis prediction scores of six ML models trained on combined data of four farms and tested on data of each individual farm separately.

Farm	ML Models	Accuracy (%)	Sensitivity (%)	Specificity (%)	Area Under Curve (%)
B	LR	94	82	94	96
	SVM	92	83	92	96
	DT	88	73	88	86
	RF	89	79	89	94
	MLP-NN	84	94	84	94
	GBDT	91	68	91	93
G	LR	97	64	97	96
	SVM	98	59	98	97
	DT	95	68	95	89
	RF	96	74	96	96
	MLP-NN	94	85	94	96
	GBDT	97	61	97	96
H	LR	91	43	91	83
	SVM	92	41	92	84
	DT	89	41	89	79
	RF	89	48	89	85
	MLP-NN	84	63	84	83
	GBDT	91	43	91	84
M	LR	93	77	93	95
	SVM	93	76	93	95
	DT	88	75	88	90
	RF	88	73	88	93
	MLP-NN	85	89	85	93
	GBDT	90	71	90	93

**Table 6:** Mastitis prediction scores of six ML models trained and tested on each individual farm’s data separately.

Farm	ML Models	Accuracy (%)	Sensitivity (%)	Specificity (%)	Area Under Curve (%)
B	LR	92	88	92	96
	SVM	92	87	92	96
	DT	95	68	95	82
	RF	97	54	98	96
	MLP-NN	94	80	94	95
	GBDT	97	61	97	93
G	LR	95	86	95	98
	SVM	96	85	96	98
	DT	96	80	96	82
	RF	96	82	96	97
	MLP-NN	93	93	93	96
	GBDT	97	77	97	94
H	LR	81	71	81	83
	SVM	82	69	82	83
	DT	87	55	88	74
	RF	91	49	91	87
	MLP-NN	73	85	73	84
	GBDT	93	45	93	85
M	LR	92	85	92	96
	SVM	93	84	93	96
	DT	90	82	90	96
	RF	90	87	90	89
	MLP-NN	86	93	86	96
	GBDT	93	81	93	96

**Table 7:** Mastitis prediction scores for six ML models trained on the data of three farms and tested on the data of the fourth farm.

Farm	ML Models	Accuracy (%)	Sensitivity (%)	Specificity (%)	Area Under Curve (%)
B	LR	88	89	88	95
	SVM	89	87	89	95
	DT	86	83	86	92
	RF	86	88	86	95
	MLP-NN	80	97	80	94
	GBDT	89	79	89	94
G	LR	97	74	97	96
	SVM	97	72	97	97
	DT	95	68	95	90
	RF	96	74	96	97
	MLP-NN	94	91	94	96
	GBDT	97	60	97	97
H	LR	89	45	90	81
	SVM	90	44	90	82
	DT	85	51	85	80
	RF	86	55	86	85
	MLP-NN	82	64	82	83
	GBDT	89	44	89	85
M	LR	90	87	90	96
	SVM	90	86	90	96
	DT	88	63	88	76
	RF	89	72	89	91
	MLP-NN	83	93	83	91
	GBDT	91	58	91	90

## Data Availability

The data that support the findings of this study are available from the corresponding author upon reasonable request.

## Abbreviations

The following abbreviations are used in this manuscript:

IMI	Intra-mammary infection
AMS	Automatic milking system
ML	Machine learning
LR	Logistic regression
SVM	Support vector machine
DT	Decision tree
RF	Random forest
MLP-NN	Multi-layer perceptron neural network
GBDT	Gradient-boosting decision tree
SCC	Somatic cell count
EC	Electrical conductivity
MF	Milk flow
MT	Milk temperature
MY	Milk yield
FM	Fat content in milk
PM	Protein content in milk
NL	Number of lactations
DIM	Days in milk
ET	Environmental temperature
EH	Environmental humidity
